# CotG Mediates Spore Surface Permeability in Bacillus subtilis

**DOI:** 10.1128/mbio.02760-22

**Published:** 2022-11-10

**Authors:** Anella Saggese, Giovanni Di Gregorio Barletta, Maria Vittoria, Giuliana Donadio, Rachele Isticato, Loredana Baccigalupi, Ezio Ricca

**Affiliations:** a Department of Biology, Federico II University, Naples, Italy; b Department of Molecular Medicine and Medical Biotechnology, Federico II University, Naples, Italy; National Institute of Child Health and Human Development (NICHD)

**Keywords:** *Bacillus subtilis*, endospores, germination, permeability, spore coat

## Abstract

Proteins and glycoproteins that form the surface layers of the *Bacillus* spore assemble into semipermeable arrays that surround and protect the spore cytoplasm. Such layers, acting like molecular sieves, exclude large molecules but allow small nutrients (germinants) to penetrate. We report that CotG, a modular and abundant component of the Bacillus subtilis spore coat, controls spore permeability through its central region, formed by positively charged tandem repeats. These repeats act as spacers between the N and C termini of the protein, which are responsible for the interaction of CotG with at least one other coat protein. The deletion but not the replacement of the central repeats with differently charged repeats affects the spore resistance to lysozyme and the efficiency of germination—probably by reducing the coat permeability to external molecules. The presence of central repeats is a common feature of the CotG-like proteins present in most *Bacillus* species, and such a wide distribution of this protein family is suggestive of a relevant role for the structure and function of the *Bacillus* spore.

## INTRODUCTION

Bacteria of the *Bacillus* genus produce (endo)spores, metabolically quiescent and extremely resistant cells (1). Spores persist almost indefinitely in environments without nutrients, under conditions of extreme temperature and pH, and in the presence of toxic chemicals and lytic enzymes (2). Although quiescent, spores constantly sense the environment and respond to the presence of nutrients by germinating and forming new vegetative cells (3).

In Bacillus subtilis, the spore core, the dehydrated cytoplasm and its membrane, is surrounded and protected by a peptidoglycan-like cortex, a proteinaceous and multilayer coat and the crust, formed by proteins and glycoproteins (2). The coat and crust form a rigid but semipermeable shell around the core, providing mechanical integrity, excluding large molecules from reaching the spore interior but allowing the transit of germinants (4). Some heterologous molecules can adsorb the spore surface and infiltrate through its surface layers without reaching the cortex or core (5). This property, which has biotechnological potential (6), depends on the physicochemical properties of both the heterologous molecules and the spore structure (7). Indeed, structurally different spores, such as, for example, those produced by B. subtilis at different temperatures, show different surface permeabilities (7).

B. subtilis spore coats produced at low or high temperature are remarkably different, with 25°C coats appearing lamellar and heavily electron dense and 42°C spores appearing granular and thick (8). The switch between the different structures is mediated by CotH, a heat-labile (8) regulatory protein known to control at least nine other coat proteins (9) and to directly interact with the major coat morphogenetic protein CotE (10). CotH is an atypical Ser/Tyr kinase that specifically phosphorylates two major outer coat proteins, CotB and CotG (11, 12). The latter is a highly abundant coat protein (13) localized in the outermost spore surface layer (14) and characterized by a modular structure with a central part formed by tandem repeats of positively charged amino acids (15).

CotG has a high net charge and is listed in the top 20 positively supercharged proteins present in data banks (16). Such a high charge is partially balanced by CotG's extensive, CotH-dependent phosphorylation (12, 17), essential for CotG assembly on the spore coat (18). Unphosphorylated, and presumably unfolded, CotG molecules do not assemble on the spore and form inclusion body-like aggregates in the mother cell cytoplasm (18). In a mutant strain lacking the kinase CotH, all CotG molecules are unphosphorylated, form massive aggregates that sequester other coat proteins, and as a consequence, cause the production of defective spores (17, 18). Such altered spores are not observed in a mutant lacking the kinase CotH but also with the central repeats of CotG deleted, indicating that the formation of cytoplasmic aggregates is due to the central part of CotG (17, 18).

The modular organization of CotG, with a central region formed by 19 tandem repeats of 6 and 7 residues, is an outcome of multiple rounds of gene elongation events of an ancestral minigene (19). Similar events presumably started from different ancestral minigenes in different *Bacillus* species, since most species contain a CotG-like protein that is not homologous to CotG of B. subtilis but shares with it the chromosomal organization, the modular structure, the presence of central repeats, and in most cases, the positive charge (19).

The focus of this report is the function of the central repeats of CotG. The analysis of mutant forms of CotG, in which the central repeats were either deleted or replaced by the central repeats of CotG of Bacillus licheniformis, suggests that the positively charged central region regulates the permeability of the spore surface, acting as a spacer between the N and C termini of the protein.

## RESULTS

### The positive charge of CotG is mostly due to its central repeats.

The net positive charge of CotG is due to the presence of 92 (47%) positively charged (K, R, and H) amino acids and only 13 (6%) negatively charged (D and E) residues. In addition, CotG has a low number (18 [9%]) of hydrophobic residues (A, G, I, L, M, P, F, W, and V), which contribute to making it an intrinsically disordered (IDP) and unstable protein (Table 1) (15). The extremely high positive net charge of CotG is partially compensated for *in vivo* by the extensive phosphorylation occurring at 15 residues, mostly localized in the central repeats of the protein, which are likely relevant also for CotG folding (see Fig. S1A in the supplemental material) (12, 17). The central repeats of CotG have a strong role in the total positive charge and in the disordered status of the entire protein. Indeed, the 19 repeats are formed by 126 amino acids (aa), with 68 (53.9%) positive residues and no negatively charged ones (Table 1).

**TABLE 1:** Structural properties of CotG of B. subtilis and B. licheniformis and the hybrid protein

Species	Protein	No. (%) of aa			Total charge^*a*^	Value by:	
		Total	Positively charged	Negatively charged		Instability index^*b*^	Unfoldability index^*c*^
B. subtilis	CotG full protein	195	92 (47.8)	13 (6.6)	+57.0	77.5	−0.722
	CotG with internal repeats deleted	126	68 (53.9)	0	+52.9	90.5	−0.908

B. licheniformis	CotG full protein	168	67 (39.8)	32 (19.0)	+20.0	26.2	−0.536
	CotG with internal repeats deleted	73	36 (49.3)	16 (21.9)	+13.8	−6.9	−0.815

B. subtilis (recombinant)	CotGHyb full protein	142	60 (42.2)	29 (20.4)	+17.8	22.5	−0.72

a At pH 7.4 (https://www.protpi.ch/Calculator/ProteinTool/).

b The instability index is an estimate of the stability of a protein. A protein with an index smaller than 40 is predicted as stable *in vitro.* The reported values were calculated with an online tool (https://web.expasy.org/protparam/).

c Disorder prediction was done by using an online tool (https://fold.proteopedia.org/cgi-bin/findex). Positive values indicate a structured polypeptide, whereas negative values indicate a disordered protein.

10.1128/mbio.02760-22.1 FIG S1 Primary sequence of CotG in (A) B. subtilis PY79 and (B) B. licheniformis ATCC 14580. Black arrows indicate the sites of phosphorylation in B. subtilis CotG. Blue and green rectangles indicate the tandem repeat modules present in CotG. (C) Schematic representation of the fusion protein GHyb. Download FIG S1, PDF file, 0.05 MB. Copyright © 2022 Saggese et al. 2022 Saggese et al. https://creativecommons.org/licenses/by/4.0/ This content is distributed under the terms of the Creative Commons Attribution 4.0 International license.

While most members of the *Bacillus* genus have a modular CotG-like protein that is positively charged and unstable (15), B. licheniformis has a weakly positive and stable CotG-like protein, as suggested by its instability index of <40 (Table 1). The central repeats of the B. licheniformis protein (Fig. S1B) contribute to these parameters, with 16 negatively charged amino acids and a negative instability index (Table 1).

In order to analyze the role of the central repeats of CotG of B. subtilis, the central repeats of B. licheniformis were used to replace the repeats of the B. subtilis protein (see Materials and Methods). The resulting hybrid protein of 142 aa (17.0 kDa) (Fig. S1C) is characterized by a modestly positive net charge and is predicted to be a stable protein (instability index of 22.5) (Table 1). The gene fusion coding for the hybrid protein (GHyb), constructed in Escherichia coli (Materials and Methods) was integrated on the chromosome of B. subtilis strain AZ604, lacking the wild-type (WT) copy of *cotG*; therefore, in the resulting strain, AZ717, GHyb was the only form of CotG present and its expression was controlled by transcription and translation signals of the B. subtilis *cotG* gene (Materials and Methods).

### The hybrid CotG is functional.

**FIG 1:**
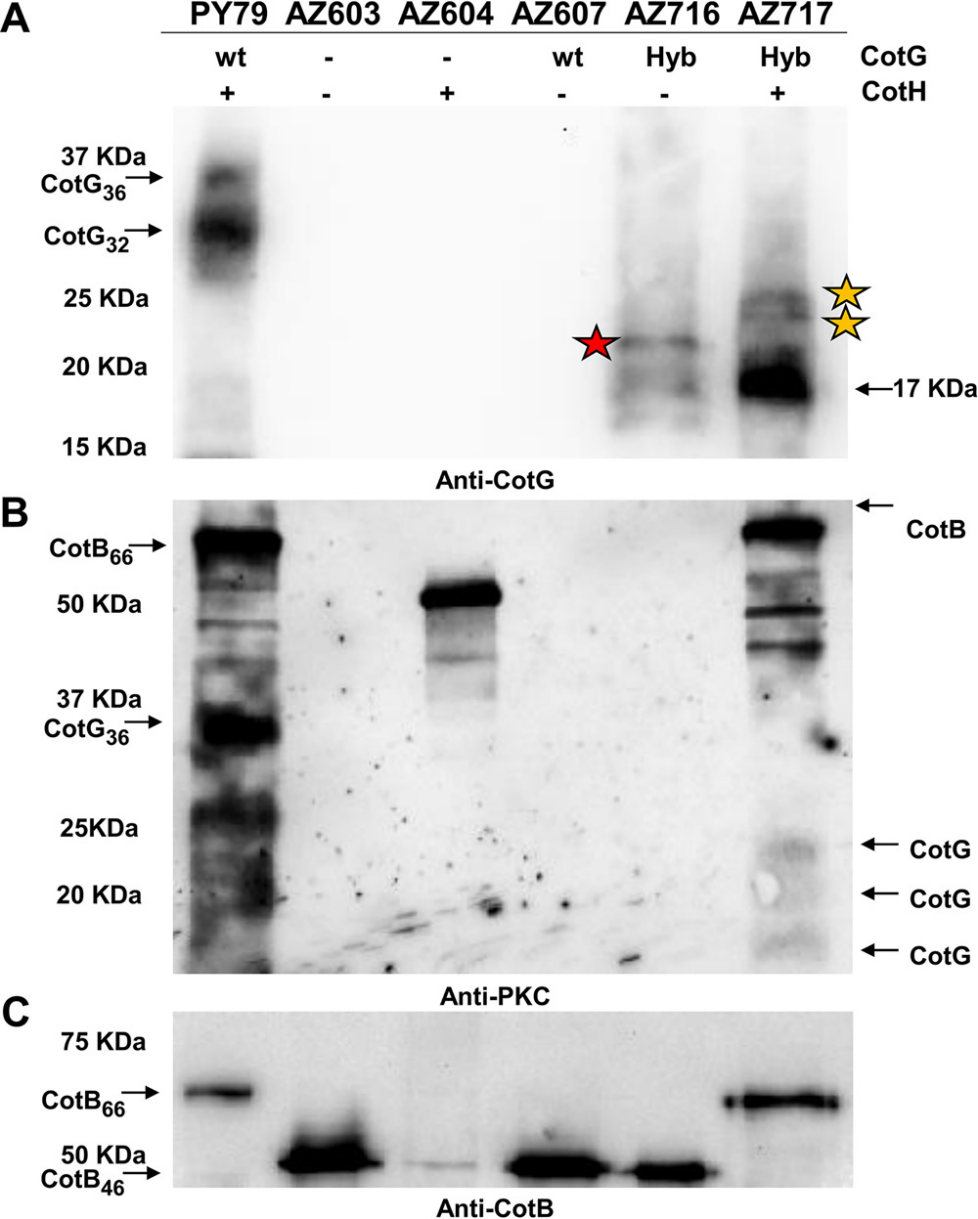
Assembly (A) and phosphorylation (B) of GHyb and (C) its effects on CotB. Western blot analyses of coat proteins extracted from purified spores of the indicated strains were performed on proteins fractionated on 12.5% SDS-PAGE gels. Electrotransferred proteins were then reacted with anti-CotG (A), anti-PKC (B), and anti-CotB (C) antibodies. The type of CotG form and the presence (+) or absence (−) of CotH are indicated. Red and yellow stars represent different GHyb forms.

To verify whether GHyb was functional, Western blotting experiments were performed on proteins extracted from purified spores of a wild-type strain and strains expressing GHyb with and without the kinase CotH. Anti-CotG antibody, raised against the N-terminal 14 residues of B. subtilis CotG (18), and anti-protein kinase C (anti-PKC) antibody, recognizing phosphorylated serine residues (18), were reacted against the extracted proteins. As previously reported, CotG was extracted from wild-type spores in two forms (CotG_36_ and CotG_32_), and both proteins were not found in extracts of spores of a strain lacking CotH (12, 20) (Fig. 1A). GHyb was instead extracted from spores independently from the presence of the kinase CotH (Fig. 1A). A faster-migrating protein corresponding to the predicted size of GHyb (17 kDa) was extracted from spores of both strains (Fig. 1A). Slower-migrating proteins were also extracted from both strains: a single protein with an apparent molecular weight of 20 kDa from a strain lacking the kinase (red star in Fig. 1A) and two proteins, both bigger than 20 kDa, in the strain expressing the kinase (yellow stars in Fig. 1A). When the anti-PKC antibody was used, no signals were observed in spores of strains lacking the kinase, indicating that all observed signals were due to CotH activity (Fig. 1B). The wild type and a *cotG* null mutant of B. subtilis showed a pattern of phosphorylated proteins consistent with that previously reported (12), with mature CotB (CotB_66_) and CotG in the wild-type strain and with the immature form of CotB (CotB_46_) in the *cotG* mutant (Fig. 1B). From the strain expressing the hybrid protein (and CotH), three proteins corresponding in size to those reacting with anti-CotG antibody in Fig. 1A and a protein corresponding in size to the mature form of CotB were observed (Fig. 1B). The additional proteins, smaller than CotB and CotG, which were also observed in Fig. 1B, were considered degradation products of CotB or CotG, since they were only recognized by anti-PKC antibody. In addition, similar proteins have been previously identified as degradation products (12).

The preliminary indication that the GHyb allowed CotB maturation (Fig. 1B) was confirmed by Western blotting with anti-CotB antibody. As previously established (12), the conversion of the immature form of CotB (CotB_46_) into its mature form (CotB_66_) requires the kinase activity of CotH and the presence of CotG. CotGHyb was able to complement the absence of CotG and allowed CotB maturation (Fig. 1C). Therefore, like a CotG form with its central repeats deleted (G^Δcentral^) (15), GHyb also allows CotB maturation, indicating that the central repeats of CotG are dispensable for this function, which is, therefore, due to the N- and C-terminal regions of the protein.

**FIG 2:**
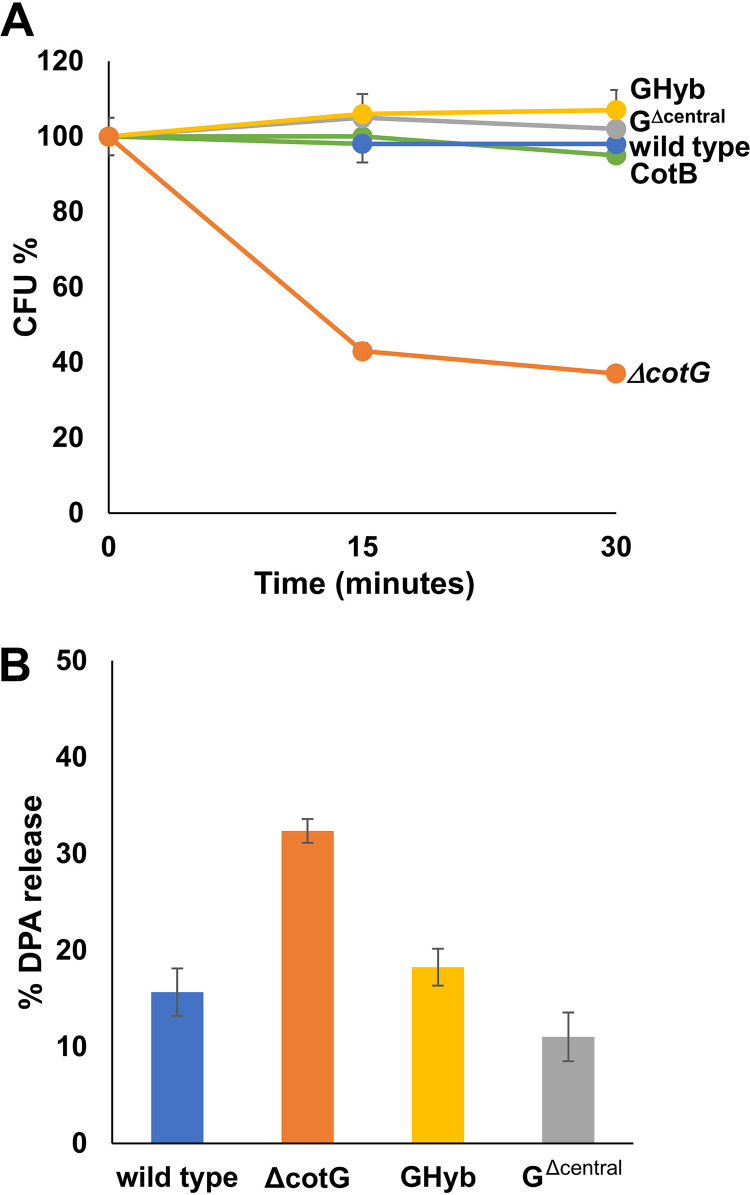
Functional analysis of spores expressing different forms of CotG. (A) Resistance to heat treatment (20 min at 80°C) and (B) DPA release (after 15 min at 100°C) of spores of the wild type (blue symbols) and mutant strains carrying a *cotG* null mutation (Δ*cotG* [orange symbols]), a *cotG* gene with the internal repeats deleted (G^Δcentral^ [gray symbols]), the hybrid *cotG* gene (GHyb [yellow symbols]), or carrying a *cotB* null mutation (CotB [green symbols]). One hundred percent is considered the amount of DPA released from spores autoclaved at 120°C for 30 min. Error bars represent standard deviations. Each percentage is the mean of results from three replicate experiments, each performed with a different prepared spore suspension.

GHyb spores were functional also in complementing the heat-sensitive phenotype of spores lacking CotG. Similar amounts of purified spores (between 0.77 × 10^8^ and 1.10 × 10^8^) were incubated at 80°C for 15 and 30 min, and their viability was assessed by plate counts. As shown in Fig. 2A, spores lacking CotG (orange line) showed decreased viability, while wild-type and GHyb spores were fully resistant. Since spores lacking CotG also lack CotB (20, 21), while GHyb spores normally assemble CotB (Fig. 1C), it is possible that the heat sensitivity observed in Fig. 2A is at least in part due to the absence of CotB. An isogenic strain carrying a null mutation in *cotB* (RH201) was then analyzed and shown to be fully resistant to the heat treatment (green line in Fig. 2A), indicating that the heat sensitivity is exclusively due to the absence of CotG and is fully complemented by GHyb. The reduced heat resistance of spores lacking CotG was consistent and most probably due to an increased release of dipicolinic acid (DPA) upon heat treatment. As shown in Fig. 2B, spores lacking CotG released 2-fold more DPA than wild-type and GHyb spores, suggesting that upon heat treatment, spores of a CotG-lacking strain are somehow leaky, release DPA, and as a consequence, show reduced viability.

### GHyb does not have negative effects on the assembly of other coat proteins and does not form cytoplasmic aggregates.

**FIG 3:**
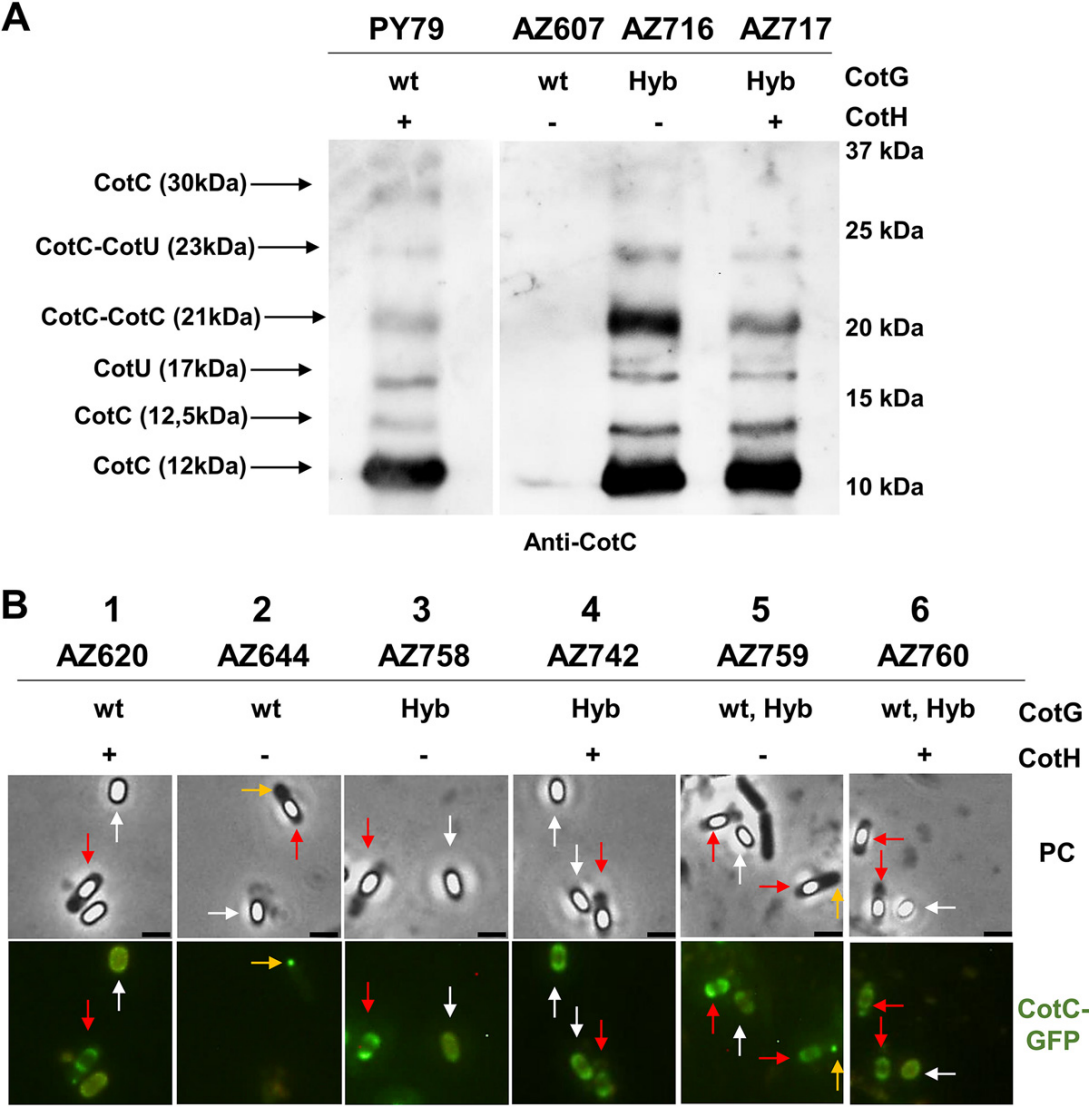
Effect of GHyb on the spore coat assembly of CotC and CotU. (A) Western blot analysis of coat proteins fractionated on 12.5% SDS-PAGE gels, electrotransferred onto a membrane, and incubated with anti-CotC antibody; (B) fluorescence microscopy analysis of spores carrying the fusion protein CotC-GFP. The type of CotG form and the presence (+) or absence (−) of CotH are indicated. Red and white arrows indicate forming and mature spores, respectively. Yellow arrows indicate CotC aggregates in the mother cell cytoplasm. Scale bar, 1 μm.

CotC and CotU, two highly homologous small coat proteins that assemble on the spore coat in multiple forms (22, 23), fail to assemble in a strain lacking CotH (17). Such an effect, due to the accumulation of unphosphorylated CotG in the mother cell cytoplasm (18), is not observed when the central repeats of CotG (G^Δcentral^) were deleted (15). Western blotting experiments showed that all forms of CotC and CotU were present in coat extracts of strains carrying GHyb as the only CotG form, independently from the presence of CotH (Fig. 3A). A fluorescence microscopy analysis of strains carrying the *cotC*::*gfp* fusion indicated proper assembly of CotC around forming and mature spores (red and white arrows in Fig. 3B, respectively), independently from the presence of CotH (Fig. 3B). Although not directly tested, CotU is likely to behave similarly based on its presence in coat extracts (Fig. 3A) and its high homology with CotC (22, 23). When GHyb was present together with a wild-type version of *cotG* (strains AZ759 and AZ760), some CotC molecules were properly assembled around forming and mature spores, while others were not assembled and found in the mother cell cytoplasm (yellow arrows in Fig. 3B). The latter result confirms that unphosphorylated CotG sequesters CotC in the mother cell cytoplasm (18) and suggests that the N- and/or the C- terminal regions of CotG (present in GHyb) are needed for CotC assembly on the spore coat. Altogether, the results of Fig. 3 indicate that, like G^Δcentral^ (15), GHyb also does not have negative effects on the assembly of CotC.

**FIG 4:**
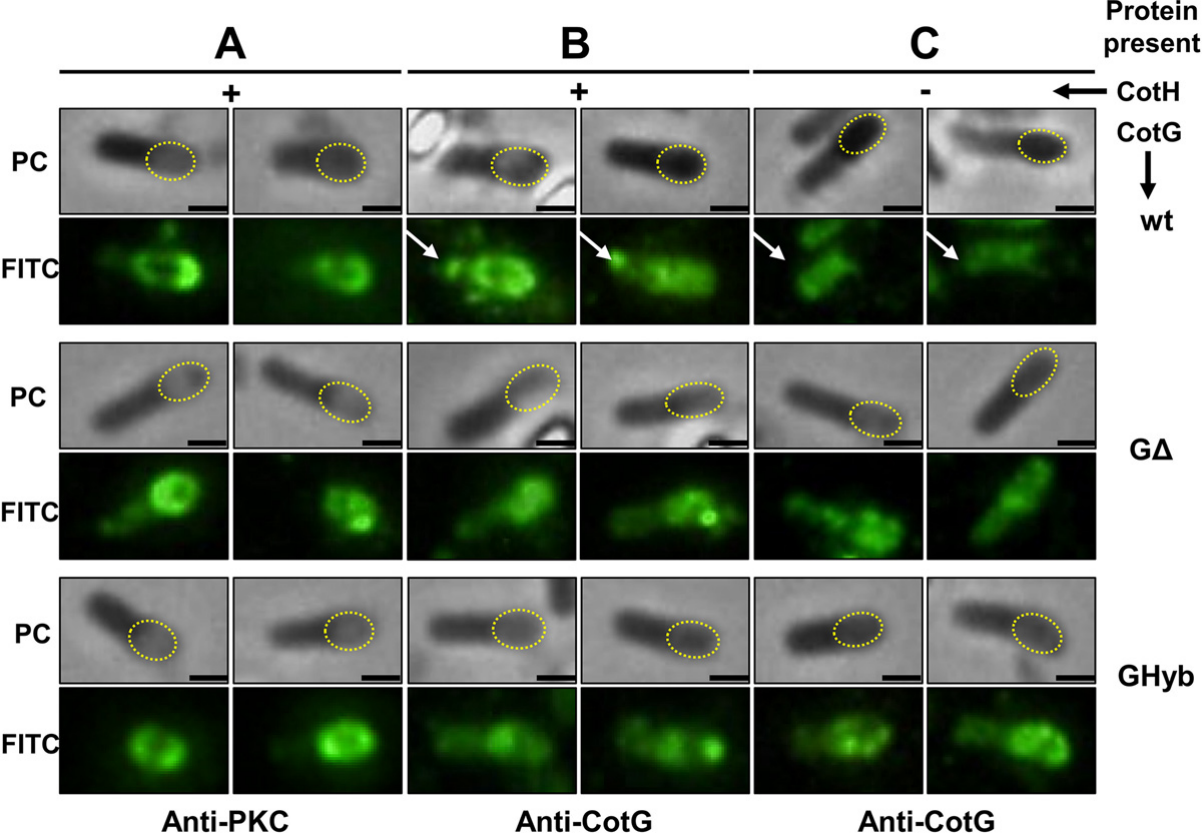
Immunofluorescence analysis of different strains carrying different forms of CotG with anti-PKC (A) and anti-CotG (B and C) antibodies in the presence (A and B) or absence (C) of CotH. In phase-contrast (PC) panels, the position of the forming spore is indicated by dotted red ovals. The exposure times were 200 ms for panel A and 500 ms for panels B and C. Scale bar, 1 μm.

This result suggests that both mutant forms of the protein do not form cytoplasmic aggregates, recently shown to sequester CotC and CotU and impair their coat assembly (18). To verify such a hypothesis, immunofluorescence microscopy experiments were performed with anti-PKC and anti-CotG antibodies. As shown in Fig. 4A and B, most GHyb and G^Δcentral^ molecules were assembled around the forming spore and only a minimal part of them were found in the mother cell cytoplasm, where they never formed the well-defined spots typical of unphosphorylated CotG of B. subtilis (arrows in Fig. 4B) (18). In a mutant lacking CotH, CotG of B. subtilis failed to assemble on the spore and massively accumulated in the mother cell (arrows in Fig. 4C) (13). In the same genetic background, the majority of the GHyb and G^Δcentral^ proteins were still able to assemble around the forming spore and the few molecules accumulated in the mother cell cytoplasm, never forming well-defined spots in the mother cell cytoplasm (Fig. 4C). Therefore, in agreement with the results of Fig. 3 and previously reported data (15), the deletion of the positively charged central repeats of CotG or their replacement with not positively charged repeats abolishes the dependency of CotG assembly on its phosphorylation and impairs the formation of CotG-dependent cytoplasmic aggregates.

### The central repeats of CotG modulate the spore functions.

**FIG 5:**
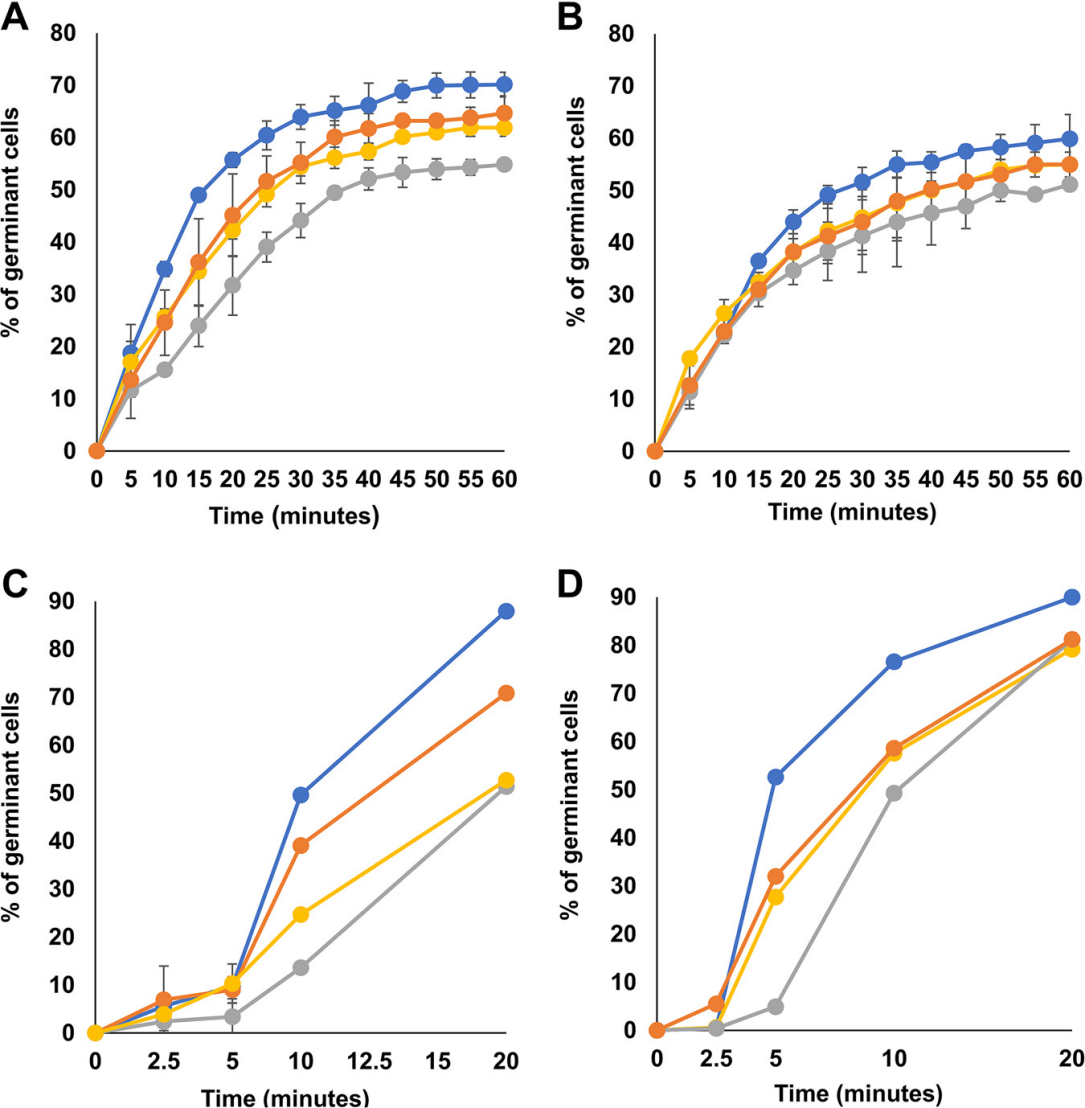
Germination efficiency of spores of the wild type (blue symbols) and mutant strains carrying a *cotG* null mutation (Δ*cotG* [orange symbols]), a *cotG* gene with the internal repeats deleted (G^Δcentral^ [gray symbols]), and the hybrid *cotG* gene (GHyb [yellow symbols]). The germination was induced using l-Ala-GFK (A and C) or l-Asn-GFK (B and D) and measured as the percentage of loss of the OD_600_ and by flow cytometry (C and D). Error bars (A and B) are based on the standard deviations of values from four independent experiments. Panels C and D report the percentage of germination obtained from flow cytometry data (Fig. S2).

The positively charged central repeats of CotG are not involved in CotB maturation (described above and see reference 15), and their only role in B. subtilis seems to be limited to the regulation of CotC-CotU assembly on the spore coat (18). However, most *Bacillus* species that have a CotG-like protein do not have CotC-CotU homologs (Table S1), suggesting that the conserved CotG protein family and their repeats might have an additional but still obscure role. To further investigate the role of CotG repeats, several spore properties were analyzed in a wild-type strain (WT) and in isogenic mutants either lacking CotG (herein indicated as Δ*cotG*) or containing G^Δcentral^ or GHyb, as described above. The efficiency of germination was assessed by using either Asn or Ala as germinants and by measuring the decrease in optical density at 590 nm (OD_590_) (Fig. 5A and B) and by flow cytometry (Fig. 5C and D and Fig. S2A and B) as previously described (24, 25). The two approaches gave similar results (Fig. 5): G^Δcentral^ spores were less efficient than those of the wild type, while Δ*cotG* and GHyb spores showed an intermediate germination efficiency. The germination defect of G^Δcentral^ spores was stronger with Asn than with Ala as the germinant, in particular at early times after the induction of germination.

10.1128/mbio.02760-22.2 FIG S2 Germination efficiency determined by flow cytometry using either Asn (A) or Ala (B) as the germinant. Similar numbers (ranging between 35,000 and 38,000) of dormant spores were considered for each strain, and fluorescence was measured before (T0) and at different time intervals after the induction of germination. For each time point of all strains, the number of germination-specific fluorescent cells (on the right side of the dotted lines) was counted, and the percentage of germination was calculated using the initial number of spores as 100. Download FIG S2, PDF file, 0.2 MB. Copyright © 2022 Saggese et al. 2022 Saggese et al. https://creativecommons.org/licenses/by/4.0/ This content is distributed under the terms of the Creative Commons Attribution 4.0 International license.

10.1128/mbio.02760-22.3 TABLE S1 Presence of *cotC*-*cotU* genes in CotG-like containing *Bacillus* species. Download Table S1, DOCX file, 0.01 MB. Copyright © 2022 Saggese et al. 2022 Saggese et al. https://creativecommons.org/licenses/by/4.0/ This content is distributed under the terms of the Creative Commons Attribution 4.0 International license.

**FIG 6:**
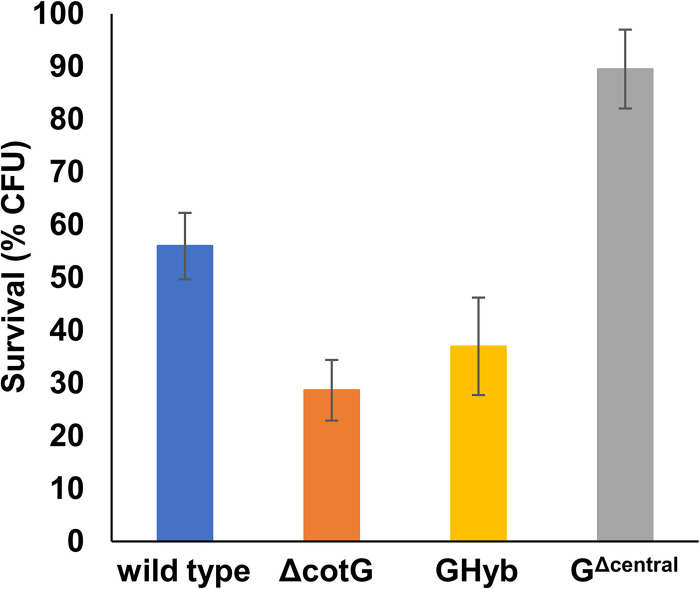
Lysozyme resistance of spores of the wild type (blue symbols) and mutant strains carrying a *cotG* null mutation (Δ*cotG* [orange symbols]), a *cotG* gene with the internal repeats deleted (G^Δcentral^ [gray symbols]), and the hybrid *cotG* gene (GHyb [yellow symbols]). Spores were incubated with lysozyme for 6 h at 37°C, and survival was estimated by CFU count. Error bars are based on the standard deviation of values from 4 independent experiments.

The resistance to lysozyme was analyzed by treating purified spores with 100 nM lysozyme (Sigma) for 360 min and measuring the cell viability on the plate (CFU). While Δ*cotG* and GHyb spores were slightly less resistant than wild-type spores, G^Δcentral^ spores were clearly more resistant (Fig. 6). Resistance to lysozyme is due to the spore coat, which acting as a sieve prevents the enzyme from reaching its target (peptidoglycan) (4). Therefore, the increased resistance of G^Δcentral^ spores suggests that the internal repeats of CotG affect the spore permeability to the lytic enzyme.

### The central repeats of CotG modulate the permeability of the spore surface.

**FIG 7:**
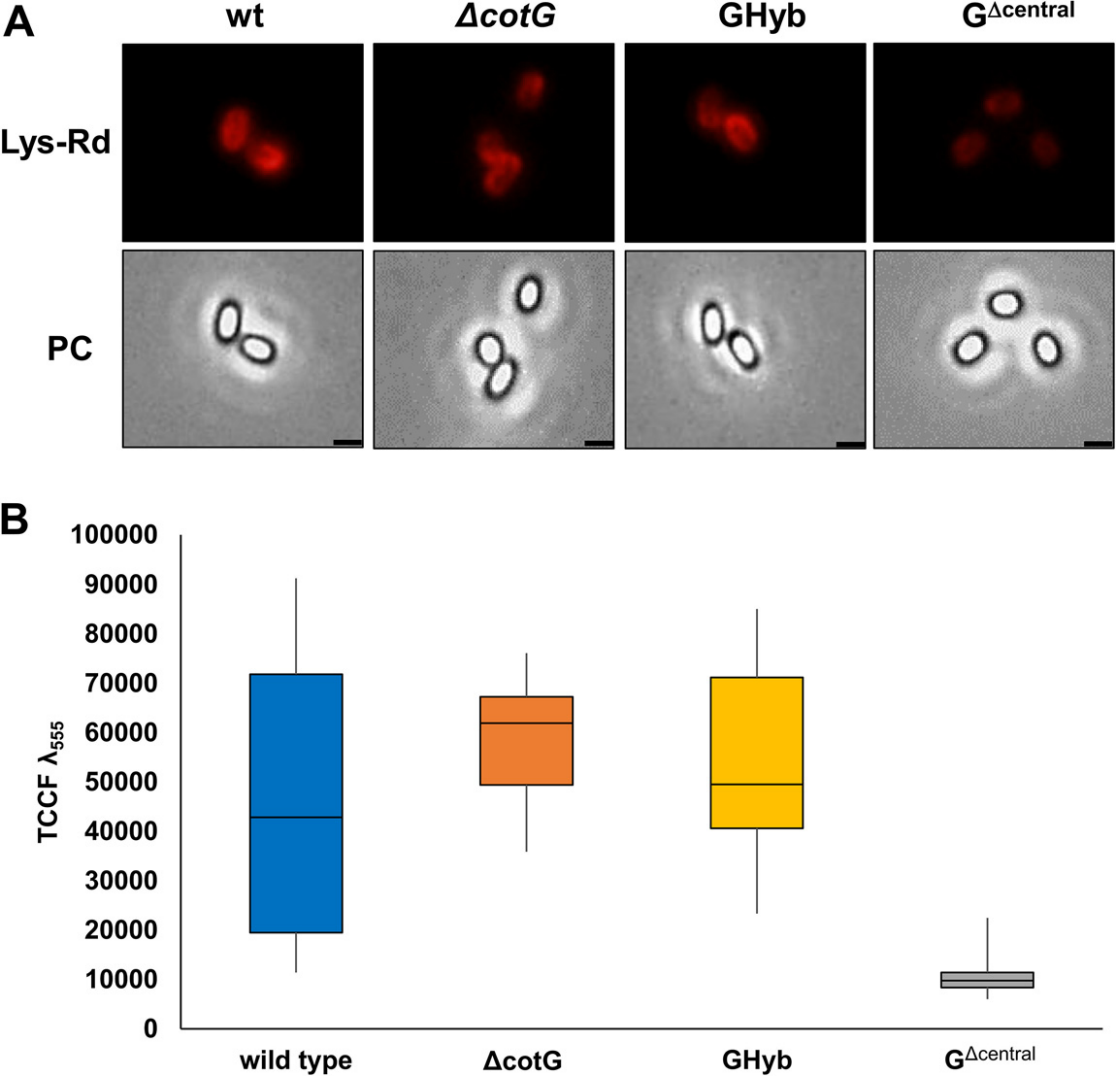
(A) Fluorescence microscopy analysis of spores of the wild type and mutant strains carrying a *cotG* null mutation (Δ*cotG*), a *cotG* gene with the internal repeats deleted (G^Δcentral^), and the hybrid *cotG* gene (GHyb) after treatment with lysozyme conjugated with rhodamine (Lys-Rd). The exposure time was 100 ms for each panel. Scale bar, 1 μm. PC, phase contrast. (B) Box plots displaying the total corrected cellular fluorescence (TCCF) for 50 different spores of each strain. The limits of each box represent the first and the third quartiles (25 and 75%), and the values outside the boxes represent the maximum and minimum values.

Heterologous proteins, when abundantly concentrated outside the spore, can cross the spore surface (5, 6). Such permeability increases the amount of protein adsorbed to the spore and varies with the chemical properties of the heterologous protein and with the coat structure (7). The adsorption of heterologous lysozyme was then used to evaluate the permeability of the surface layers of wild-type, Δ*cotG*, GHyb, and G^Δcentral^ spores. Lysozyme was fluorescently labeled with rhodamine (Lys-Rd) as previously described (7), and 10 mM labeled protein used to treat 5.0 × 10^8^ purified spores of the four strains. The reactions were carried out for 1 h at 37°C in 50 mM sodium citrate buffer (pH 4.0) as previously described for other heterologous proteins (26). Spores were collected by centrifugation and analyzed by fluorescence microscopy and flow cytofluorometry as previously described (7). The labeled lysozyme adsorbed to all four types of spores, and the intensity of the fluorescence signal was weaker with G^Δcentral^ spores than with all other spores (Fig. 7A). The relative fluorescence signals were analyzed by the ImageJ software (NIH) as previously reported (5) and appeared high and heterogeneous in wild-type spores, while the signals were low and homogeneous in G^Δcentral^ spores (Fig. 7B). Fluorescence signals in Δ*cotG* and GHyb spores were slightly stronger and less heterogeneous than those of wild-type spores. The fluorescence heterogeneity observed with wild-type spores, and in part with Δ*cotG* and GHyb spores, is due to a combination of highly and weakly fluorescent spores, suggestive of a porous spore surface easily crossed by the labeled lysozyme molecules. As a consequence, different numbers of fluorescent molecules localize at different positions within the coat (6), producing signals of heterogeneous intensities. The fluorescence homogeneity, as well as the low red emission intensity, observed with G^Δcentral^ spores is instead suggestive of a compact, less porous surface that cannot be easily crossed. As a consequence, all lysozyme molecules localize in the same position and produce homogeneous fluorescent signals.

**FIG 8:**
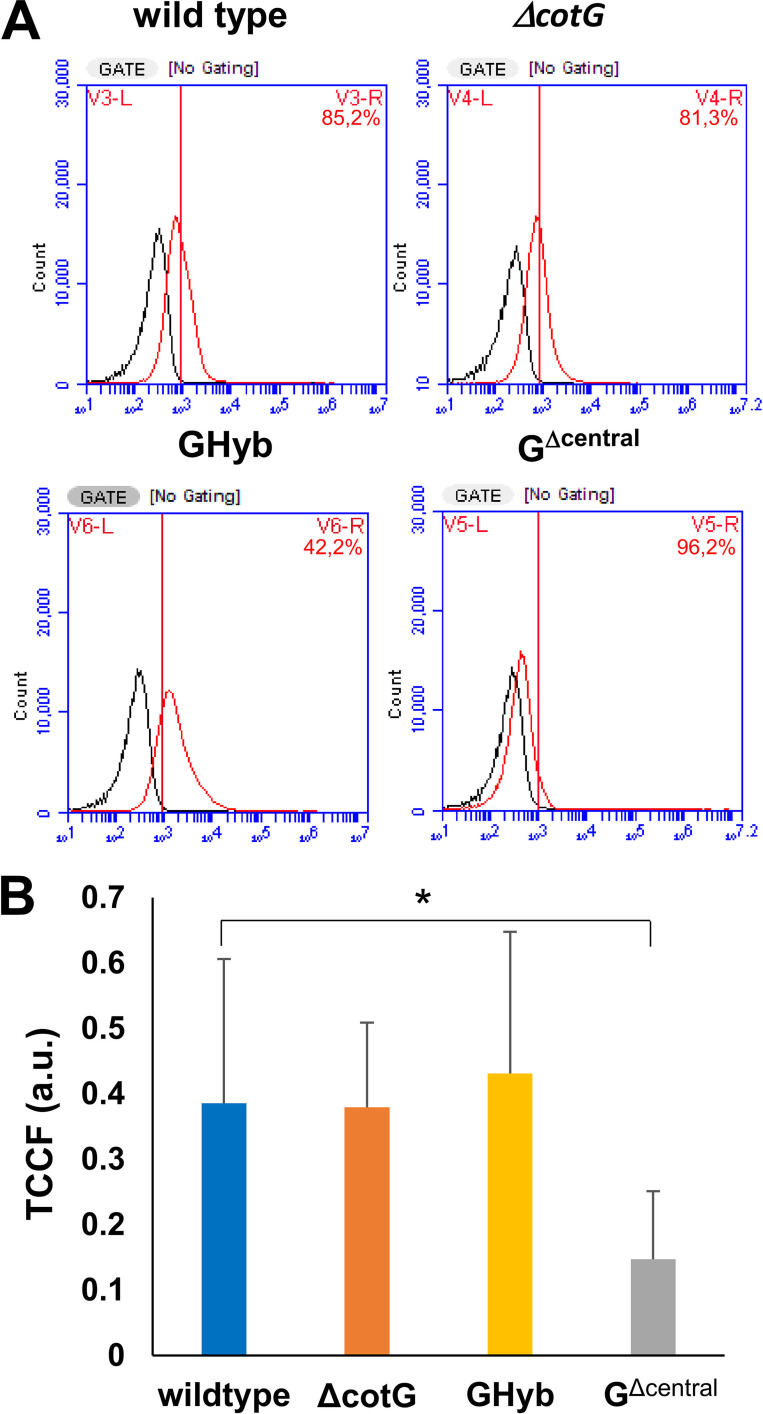
(A) Flow cytometry analysis of 10,000 spores of the wild type and mutant strains carrying a *cotG* null mutation (Δ*cotG*), a *cotG* gene with the internal repeats deleted (G^Δcentral^), and the hybrid *cotG* gene (GHyb) after treatment with lysozyme conjugated with rhodamine (Lys-Rd). The black curves represent the nonspecific fluorescence signal of the spores (no Lys-Rd), whereas the red curves represent the fluorescence signal after treatment with Lys-Rd. (B) Quantitative analysis of the fluorescence of over 50 spores by the ImageJ software. The *y* axis describes the total corrected cellular fluorescence (TCCF) value. The fluorescent intensity threshold is 1 × 10^3^ (red line). a.u., arbitrary units.

The fluorescence signals were quantitatively analyzed in duplicate on 100,000 spores each by flow cytometry. The analysis confirmed the fluorescence microscopy results and indicated that the only statistically significant difference was between wild-type and G^Δcentral^ spores (*P* < 0.001) (Fig. 8).

**FIG 9:**
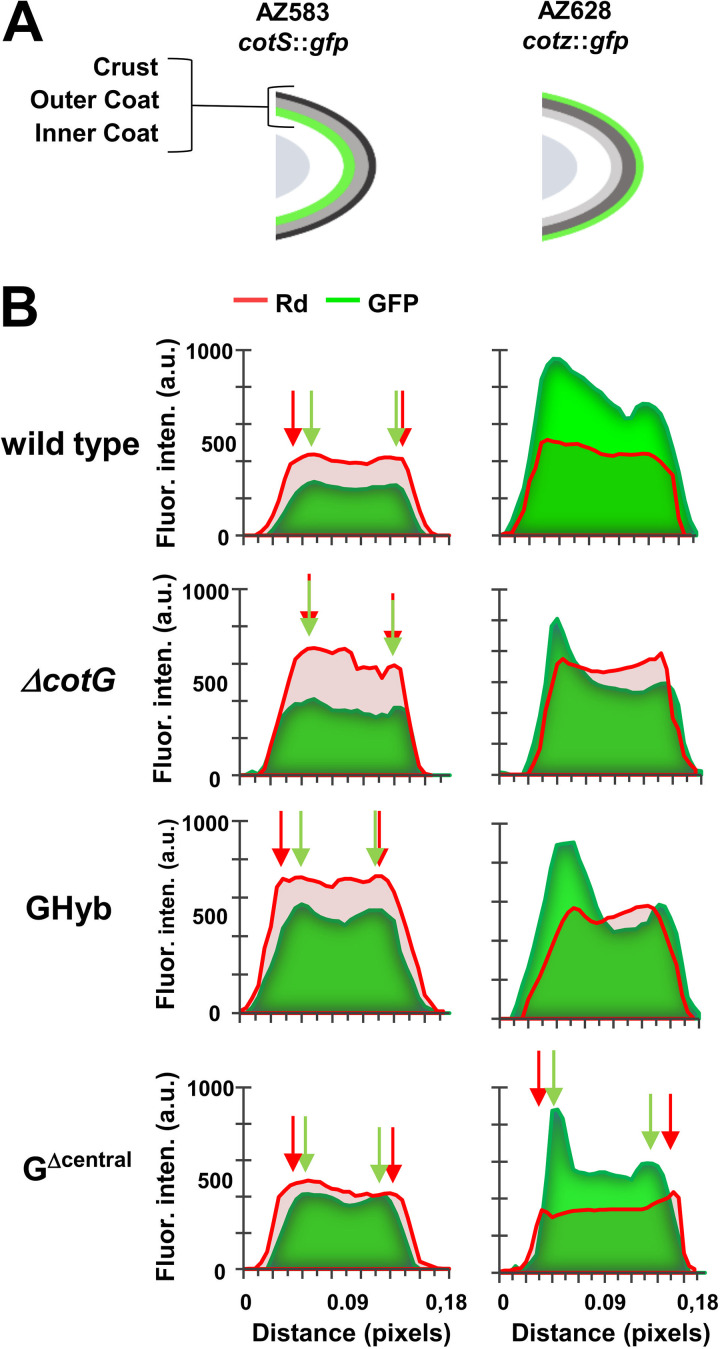
(A) Schematic representation of the localization of GFP in the spore coat of strains carrying GFP fused to inner coat (*cotS::gfp*), outer coat (*cotC::gfp*), or crust (*cotZ::gfp*) proteins; (B) plots of green (GFP) and red (Lys-Rd) fluorescence intensities along the long axis of spores of the strains indicated in panel A. Green and red arrows indicate peaks of GFP and Rd fluorescence intensities and are represented only when the Rd fluorescence signals colocalize or Rd signal is more external than the GFP signal. One pixel corresponds to 1.18 nm. a.u., arbitrary units.

To support the hypothesis that the reduced adsorption of G^Δcentral^ spores was due to a reduced permeability to lysozyme, the gene coding for the green fluorescent protein (GFP) fused to B. subtilis genes coding for proteins known to localize in the inner coat (CotS) (14, 27) or in the crust (CotZ) (14) were moved by chromosomal DNA-mediated transformation into wild-type, CotG, GHyb, and G^Δcentral^ strains. Purified spores of the resulting strains (see Materials and Methods) were treated with the rhodamine-labeled lysozyme and observed by fluorescence microscopy. More than 80 free spores from each strain were used to measure the intensity of GFP and Lys-Rd on the spore long axis (indicated by the *x* axis in Fig. 9) by using ImageJ software as previously described (5). Averages of red and green fluorescence intensities of the various strains are plotted in Fig. 9. Although the size of the observed structures is below the resolution limit of the fluorescence microscope, the averaged results suggested a different permeability to the lytic enzyme for the spores of the various strains. The red fluorescence signal of the labeled lysozyme was always external to the inner coat (*cotS*::*gfp*) and internal to the crust (*cotZ*::*gfp*) for spores of all strains but G^Δcentral^ spores (red and green arrows in Fig. 9).

## DISCUSSION

**FIG 10:**
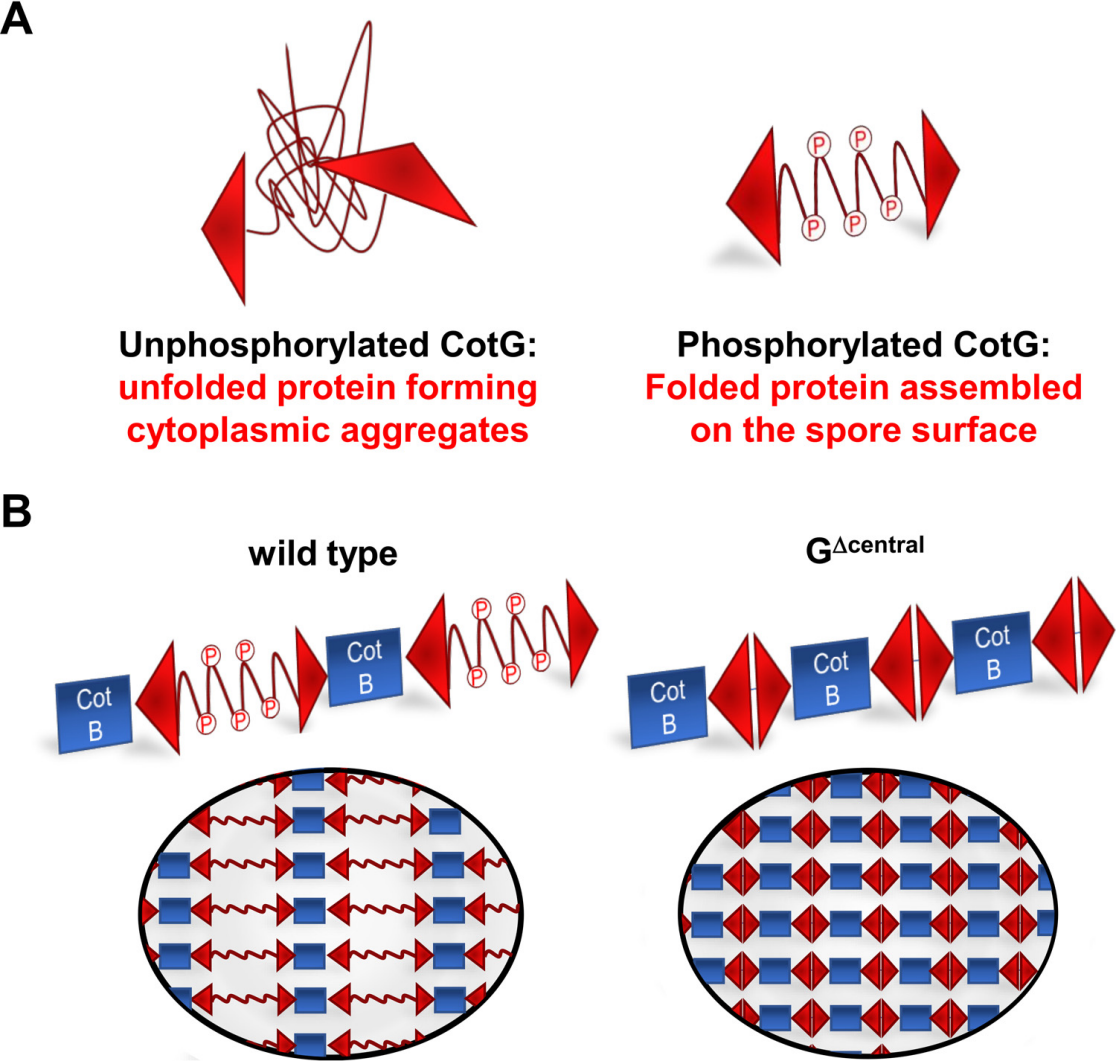
(A) Schematic representation of unphosphorylated and phosphorylated forms of CotG protein; (B) working model of the role of the central repeats of CotG in controlling spore permeability.

The CotG-like protein family is conserved in sporeformers of the *Bacillus* genus. Members of the family do not have a homologous primary sequence but share a similar modular structure with a central part formed by several repeats containing, in most cases, a high percentage of positively charged amino acids (15). In B. subtilis, CotG is present in sporulating cells as a phosphorylated protein that is readily assembled around the forming spore and as a presumably unfolded unphosphorylated protein that accumulates in the mother cell cytoplasm, where it forms aggregates able to sequester other coat proteins (i.e., CotC and CotU) (18). On the spore, CotG is localized in the outermost layer (14) and interacts with CotB, and such interaction is strictly required for the CotH-dependent conversion of the immature form of CotB (CotB_46_) into its mature form (CotB_66_) (12, 21). Results of this study clarify that when the central repeats of CotG are replaced by repeats that are not as positively charged (GHyb), the assembly of the protein on the spore surface no longer depends on its phosphorylation, pointing to the massive CotH-dependent phosphorylation of CotG as an essential step needed to balance the high positive charge of the protein and probably to allow its proper folding as schematically represented in Fig. 10A.

When the internal repeats of CotG are either lacking (G^Δcentral^) or have been replaced (GHyb), the protein is able to interact with CotB (Fig. 1) and allows CotC assembly (Fig. 3B), indicating that the central repeats are not involved in such interaction and may have different functions. Although we cannot rule out the possibility that the deletion of the central repeats of CotG alters the coat structure, causing pleiotropic effects, the reduced efficiency of germination and the increased resistance to lysozyme together point to the central repeats of CotG as a modulator of the permeability of the spore surface. Such a conclusion is also indirectly supported by the reduced adsorption of lysozyme to the spore surface and by its localization outside the spore crust in a strain lacking the central repeats of CotG. A working model developed on the basis of the reported results hypothesizes that the N and C termini of CotG, involved in the interaction with another coat protein (CotB), are separated by the internal repeats acting as spacers (Fig. 10B). When the central repeats of CotG are deleted (G^Δcentral^), the N and C termini are close to each other, the connection with CotB (and possibly with other coat proteins) is tight, and the spore is not permeable and highly resistant to lysozyme (Fig. 10B). When the central repeats of CotG are replaced by repeats that are not as positively charged (GHyb), the permeability of the spore surface is more similar to that of wild-type spores than G^Δcentral^ spores, indicating that the presence of the repeats allows a distance between the N and C termini and, as a consequence, the formation of a less compact and more permeable spore surface. When CotG is totally lacking, the interaction with CotB cannot occur, causing the formation of a completely different spore surface. Indeed, when CotG (and therefore CotB) is lacking (12) or when the protein’s abundance is strongly reduced (8), the spore surface has a totally different structure. Several attempts to purify CotG after overexpression in heterologous hosts have been so far unsuccessful, suggesting the toxicity of the unphosphorylated protein. Low levels of CotG were produced in E. coli also when the kinase CotH was coexpressed (12). However, such levels were not sufficient to purify enough protein for structural studies, impairing the possibility to propose a more definite model so far.

## MATERIALS AND METHODS

### Bacterial strains and molecular procedures.

Strains are listed in Table S2 in the supplemental material. Plasmid amplification for nucleotide sequencing, subcloning experiments, and transformation of Escherichia coli competent cells were performed with E. coli strain DH5α (28). Bacterial strains were transformed by previously described procedures: CaCl_2_-mediated transformation of E. coli competent cells (28) and two-step transformation of B. subtilis (29). DNA ligation, isolation of plasmids, and restriction digestions were performed by using standard methods (28). Chromosomal DNA extraction of B. subtilis and B. licheniformis was carried out as described previously (29). The replacement of the antibiotic resistance gene cassettes was performed as described previously (30).

10.1128/mbio.02760-22.4 TABLE S2 *Bacillus* strains used in this study. Download Table S2, DOCX file, 0.02 MB. Copyright © 2022 Saggese et al. 2022 Saggese et al. https://creativecommons.org/licenses/by/4.0/ This content is distributed under the terms of the Creative Commons Attribution 4.0 International license.

### B. subtilis strain construction.

The gene fusion coding for GHyb was constructed in E. coli. The 5′ (including the promoter region) and 3′ portions of *cotG* of B. subtilis were PCR amplified using B. subtilis PY79 as a template and oligonucleotide pairs G1-H19/Glich1 (for 5′ region) and G3-Glich4/X2 (Table S3). DNA coding for the internal repeats of the CotG-like of B. licheniformis was PCR amplified by using chromosomal DNA of strain ATCC 14580 as a template and the oligonucleotide pairs Glich2/Glich3 (Table S3). The partially overlapping PCR products were fused by using the gene SOEing (splicing by overlap extension) technique (31) to obtain a fusion DNA fragment that was cloned into the pGEM-T Easy vector (Promega). The cloned fragment was analyzed by nucleotide sequencing. The fusion fragment was then digested with the restriction enzymes BamHI and HindIII and inserted into pDG1731, an integrative vector commonly used to integrate cloned genes at the *thrC* gene (31), previously digested with the same enzymes. The obtained plasmid, pOS16, was linearized with ScaI and used to transform competent cells of PY79 to yield strain AZ715 (*thrC*::*cotGHyb*). The gene fusion was then moved by chromosomal DNA-mediated transformation into competent cells of strains AZ603 and AZ604 (Table S2) to yield strains AZ716 (Δ*cotG* Δ*cotH thrC*::*GHyb*) and AZ717 (Δ*cotG* Δ*cotH amyE*::*cotG*_stop_*cotH thrC*::*GHyb*).

10.1128/mbio.02760-22.5 TABLE S3 Oligonucleotides used in this study. Download Table S3, DOCX file, 0.01 MB. Copyright © 2022 Saggese et al. 2022 Saggese et al. https://creativecommons.org/licenses/by/4.0/ This content is distributed under the terms of the Creative Commons Attribution 4.0 International license.

The chloramphenicol resistance gene cassette of strains RH238 (*cotC*::*gfp*), AZ644 (*cotS*::*gfp*), and AZ573 (*cotZ*::*gfp*) was replaced with tetracycline resistance gene cassette by transformation with plasmid p::TC (27), obtaining AZ640 (*cotC*::*gfp*), AZ583 (*cotS*::*gfp*), and AZ628 (*cotZ*::*gfp*), respectively. Chromosomal DNA of AZ583 (*cotS:gfp*) was used to transform compentent cells of strains AZ604, AZ613, AZ717 to yield strains AZ681 (Δ*cotG* Δ*cotH amyE*::*cotGstopcotH cotS*::*gfp*), AZ694 (ΔcotG Δ*cotH amyE*::*cotGstopcotH thrC*::*cotGΔCentral cotS*:*gfp*) and AZ752 (Δ*cotG* Δ*cotH amyE*::*cotGstopcotH thrC*::*GHyb cotS*:*gfp*) respectively. Chromosomal DNA of AZ628 (*cotZ:gfp*) was used to transform compentent cells of strains AZ604, AZ613, AZ717 to yield strains AZ682 (Δ*cotG* Δ*cotH amyE*::*cotGstopcotH cotZ*::*gfp*), AZ695 (Δ*cotG* Δ*cotH amyE*::*cotGstopcotH thrC*::*cotG*Δ*Central cotZ:gfp*) and AZ751 (Δ*cotG ΔcotH amyE*::*cotGstopcotH thrC::GHyb cotZ:gfp*) respectively. Chromosomal DNA of AZ640 (cotC:gfp) was used to transform competent cells of AZ607, AZ717, AZ716, AZ715 to obtain AZ623 (ΔcotG ΔcotH::neo amyE::cotG cotC::gfp), AZ742 (Δ*cotG* *ΔcotH*::*neo* *amyE*::*cotGstopcotH thrC*::*GHyb cotC*::*gfp*) AZ758 (Δ*cotG* Δ*cotH thrC*::*GHyb cotC*::*gfp*) and AZ760 (*thrC*::*cotGHyb cotC*::*gfp*), respectively.

The spectinomycin resistance gene cassette of strain ER220 (Δ*cotH*::*spec*) was replaced with the neomycin resistance gene cassette by transformation with plasmid p::Neo (27), obtaining AZ664 (Δ*cotH*::*neo*). Then, chromosomal DNA of AZ664 was used to transform competent cells of strain AZ715 (*thrC*::*cotGHyb*) to obtain AZ757 (Δ*cotH*::*neo thrC*::*cotGHyb*). Finally, chromosomal DNA of AZ640 (*cotC*::*gfp*) was used to transform competent cells of AZ757 to obtain AZ759 (Δ*cotH*::*neo thrC*::*cotGHyb cotC*::*gfp*) (Table S2).

### Spore purification, coat protein extraction, and Western blot analysis.

Sporulation of all strains was induced by growing cells in Difco sporulation medium (DSM) at 37°C with vigorous shaking. After 30 h, spores were analyzed under the light microscope, collected by centrifugation, washed four times with distilled water, and purified as described previously (32). In particular, spores were purified by a lysozyme treatment (29) when they were prepared for coat protein extraction and by overnight incubation in H_2_O at 4°C to lyse residual sporangial cells (25, 32) when prepared for functional analysis or lysozyme adsorption. Spore purity was checked by microscopic inspection, and the preparation was considered pure when less than 5% of sporangia were present. Spore coat proteins were extracted from a suspension of 1 × 10^9^ spores by SDS-dithiothreitol (DTT) treatment (29). The extracted proteins were quantified with a Bio-Rad DC protein assay kit (Bio-Rad), and 20 μg of total proteins was fractionated on 12.5 or 15% polyacrylamide SDS-PAGE gels. For Western blotting, SDS-PAGE gels were electrotransferred on a nitrocellulose filter (Bio-Rad), and filters were reacted with polyclonal antibodies. Anti-CotC, anti-CotB, and anti-CotG antibodies were diluted 1:7,000, while commercial anti-PKC antibody (Cell Signaling Technology) was diluted 1:10.000. Then, a horseradish peroxidase (HRP)-conjugated anti-rabbit secondary antibody (Santa Cruz) was used to recognize specific bands that were visualized by the SuperSignal West Pico chemiluminescence (Pierce) method as specified by the manufacturer.

### Physiological analysis.

**(i) Heat resistance.** Suspensions of 1.0 × 10^7^ spores were incubated at 80°C for 0, 15, and 30 min, and then the suspensions were serially diluted and plated on LB agar (2%) plates and incubated overnight at 37°C for CFU count.

**(ii) DPA release.** Suspensions of 1.0 × 10^9^ spores were incubated at 100°C for 15 min or autoclaved at 120°C for 30 min and centrifuged for 10 min at 13,000 × *g*, and then 150 μL of supernatant was placed in a 96-well microtiter plate with 150 μM TbCl_3_ in 400 mM sodium acetate buffer (pH 5) and analyzed with a BioTek Synergy H4 microplate reader. One hundred percent was considered the amount of DPA released from spores autoclaved at 120°C for 30 min (total DPA content). Spores of all strains contained similar amounts of DPA ranging between 108 and 146 μM. For all samples, the DPA content was measured as described previously (8).

**(iii) Lysozyme resistance.** Spores were prepared as described above, omitting the lysozyme step and eliminating vegetative cells by heat treatment (10 min at 80°C). Purified spores were heat treated (20 min at 80°C), suspended in 10 mM Tris-HCl (pH 7.0) buffer containing lysozyme (0.01 mg/mL), and incubated at 37°C for 0 h and 6 h. Finally, the suspension was diluted and plated on LB agar (2%) and incubated overnight at 37°C for the CFU count.

**(iv) Germination efficiency.** Purified spores were heat activated (30 min at 70°C), and germination was induced by adding the indicated germinants. Germination was measured by OD_590_ decrease (25) and by flow cytometry (24, 25) by using as the germinant 10 mM l-asparagine or 10 mM l-alanine in a buffer containing 10 mM Tris-HCl (pH 8.0) and a buffer containing 1 mM fructose, 1 mM glucose, and 10 mM KCl. The flow cytometry approach was based on the use of a nucleic acid stain, SYTO 16, that was added to spores before the induction of germination and the fluorescence followed by a BD Accuri C6 flow cytometer. Since nucleic acids of dormant spores are not accessible to SYTO 16, while those of germinating spores are efficiently stained (24), different numbers of highly fluorescent cells are indicative of different levels of germination (24).

### Fluorescence and immunofluorescence microscopy.

Fluorescence analyses were carried out using an Olympus BX51 fluorescence microscope fitted with a 100× objective UPlanF1. Fluorescein isothiocyanate (FITC) (U-MNIB2) and tetramethyl rhodamine isocyanate (TRITC) (U-MWIG2) filters were used to detect the green and red fluorescence signals, respectively. Images were captured using an Olympus DP70 digital camera equipped with an Olympus U-CA magnification changer and processed with Image Analysis software (Olympus) for minor adjustments of brightness, contrast, and color balance and for creation of merged images. Fluorescence intensities and the distance between two fluorescent peaks were measured using unadjusted merged images with ImageJ processing software (version 1.48; NIH) as previously described (5). One pixel corresponds to 1.18 nm in our detection system. ImageJ was also used to draw an outline around 80 spores for each strain, prior to area, integrated density, and the mean fluorescence measurements being recorded, together with several adjacent background readings. The total corrected cellular fluorescence (TCCF) was calculated by subtracting (area of selected cell × mean fluorescence of background readings) from integrated density values.

Immunofluorescence experiments were performed as described previously (18). In brief, 1 mL of sporulating cells was fixed for 1 h at room temperature in 80% methanol and then washed and incubated overnight at 4°C in 100% methanol. Fixed sporangia were permeabilized by incubating samples with a solution containing GTE (5% glucose, 0.01 M EDTA [pH 8.0], 20 mM Tris-HCl [pH 7.5]) plus 0.2 mg/mL lysozyme and rapidly placed on a coverslip previously treated for 30 s with poly-l-lysine. The coverslips were air dried, and cells were pretreated with 1% (wt/vol) dried milk in phosphate-buffered saline (PBS [pH 7.4]) prior to 2 h of incubation at 4°C with the monoclonal anti-CotC primary antibody (raised in rabbit; 200-fold dilution). After four washes with PBS, the samples were incubated with a 64-fold-diluted anti-rabbit secondary antibody, conjugated with fluorescein isothiocyanate (FITC) (Bethyl Laboratories) for 2 h at room temperature in the dark. After four washes, the coverslips were mounted onto microscope slides, one drop of PBS was added, and the samples were analyzed by fluorescence microscopy (18). Sporangia with only addition of the secondary FITC-conjugated antibody were used as a control of the specificity of this technique.

### Lysozyme labeling and treatment of B. subtilis spores.

Lysozyme was fluorescently labeled with rhodamine as previously described (5). Labeled lysozyme (Lys-Rd) was used to treat spores. In brief, 10 mM Lys-Rd was added to a suspension of 5.0 × 10^8^ spores in 50 mM sodium citrate pH 4.5 in a final volume of 200 μL. After 1 h of incubation at 25°C, the binding mixtures were washed and centrifuged (10 min at 13,000 × *g*) to fractionate adsorbed spores (pellet) from unbound protein (supernatant). Collected spores were analyzed by fluorescence microscopy or flow cytometry as described in reference 7.

## References

[1] Riley EP, Schwarz C, Derman AI, Lopez-Garrido J. 2020. Milestones in *Bacillus subtilis* sporulation research. Microb Cell 8:1–16. doi:10.15698/mic2021.01.739. 33490228 PMC7780723

[2] McKenney PT, Driks A, Eichenberger P. 2013. The *Bacillus subtilis* endospore: assembly and functions of the multilayered coat. Nat Rev Microbiol 11:33–44. doi:10.1038/nrmicro2921. 23202530 PMC9910062

[3] Christie G, Setlow P. 2020. *Bacillus* spore germination: knowns, unknowns and what we need to learn. Cell Signal 74:109729. doi:10.1016/j.cellsig.2020.109729. 32721540

[4] Lai E-M, Phadke ND, Kachman MT, Giorno R, Vazquez S, Vazquez JA, Maddock JR, Driks A. 2003. Proteomic analysis of the spore coats of *Bacillus subtilis* and *Bacillus anthracis*. J Bacteriol 185:1443–1454. doi:10.1128/JB.185.4.1443-1454.2003. 12562816 PMC142864

[5] Donadio G, Lanzilli M, Sirec T, Ricca E, Isticato R. 2016. Localization of a red fluorescence protein adsorbed on wild type and mutant spores of *Bacillus subtilis*. Microb Cell Fact 15:153. doi:10.1186/s12934-016-0551-2. 27609116 PMC5016992

[6] Ricca E, Baccigalupi L, Isticato R. 2021. Spore-adsorption: mechanism and applications of a non-recombinant display system. Biotechnol Adv 47:107693. doi:10.1016/j.biotechadv.2020.107693. 33387640

[7] Petrillo C, Castaldi S, Lanzilli M, Saggese A, Donadio G, Baccigalupi L, Ricca E, Isticato R. 2020. The temperature of growth and sporulation modulates the efficiency of spore-display in *Bacillus subtilis*. Microb Cell Fact 19:185. doi:10.1186/s12934-020-01446-6. 33004043 PMC7528486

[8] Isticato R, Lanzilli M, Petrillo C, Donadio G, Baccigalupi L, Ricca E. 2020. *Bacillus subtilis* builds structurally and functionally different spores in response to the temperature of growth. Environ Microbiol 22:170–182. doi:10.1111/1462-2920.14835. 31713316

[9] Kim H, Hahn M, Grabowski P, McPherson DC, Otte MM, Wang R, Ferguson CC, Eichenberger P, Driks A. 2006. The *Bacillus subtilis* spore coat protein interaction network. Mol Microbiol 59:487–502. doi:10.1111/j.1365-2958.2005.04968.x. 16390444

[10] Isticato R, Sirec T, Vecchione S, Crispino A, Saggese A, Baccigalupi L, Notomista E, Driks A, Ricca E. 2015. The direct interaction between two morphogenetic proteins is essential for spore coat formation in *Bacillus subtilis*. PLoS One 10:e0141040. doi:10.1371/journal.pone.0141040. 26484546 PMC4618286

[11] Nguyen KB, Sreelatha A, Durrant ES, Lopez-Garrido J, Muszewska A, Dudkiewicz M, Grynberg M, Yee S, Pogliano K, Tomchick DR, Pawłowski K, Dixon JE, Tagliabracci VS. 2016. Phosphorylation of spore coat proteins by a family of atypical protein kinases. Proc Natl Acad Sci USA 113:E3482–E3491. doi:10.1073/pnas.1605917113. 27185916 PMC4922149

[12] Freitas C, Plannic J, Isticato R, Pelosi A, Zilhão R, Serrano M, Baccigalupi L, Ricca E, Elsholz AKW, Losick R, O Henriques A. 2020. A protein phosphorylation module patterns the *Bacillus subtilis* spore outer coat. Mol Microbiol 114:934–951. doi:10.1111/mmi.14562. 32592201 PMC7821199

[13] Sacco M, Ricca E, Losick R, Cutting S. 1995. An additional GerE-controlled gene encoding an abundant spore coat protein from *Bacillus subtilis*. J Bacteriol 177:372–377. doi:10.1128/jb.177.2.372-377.1995. 7814326 PMC176600

[14] Imamura D, Kuwana R, Takamatsu H, Watabe K. 2011. Proteins involved in formation of the outermost layer of *Bacillus subtilis* spores. J Bacteriol 193:4075–4080. doi:10.1128/JB.05310-11. 21665972 PMC3147665

[15] Saggese A, Isticato R, Cangiano G, Ricca E, Baccigalupi L. 2016. CotG-like modular proteins are common among spore-forming bacilli. J Bacteriol 198:1513–1520. doi:10.1128/JB.00023-16. 26953338 PMC4859607

[16] Ma C, Malessa A, Boersma AJ, Liu K, Herrmann A. 2020. Supercharged proteins and polypeptides. Adv Mater 32:e1905309. doi:10.1002/adma.201905309. 31943419

[17] Saggese A, Scamardella V, Sirec T, Cangiano G, Isticato R, Pane F, Amoresano A, Ricca E, Baccigalupi L. 2014. Antagonistic role of CotG and CotH on spore germination and coat formation in *Bacillus subtilis*. PLoS One 9:e104900. doi:10.1371/journal.pone.0104900. 25115591 PMC4130616

[18] Di Gregorio Barletta G, Vittoria M, Lanzilli M, Petrillo C, Ricca E, Isticato R. 2022. CotG controls spore surface formation in response to the temperature of growth in *Bacillus subtilis*. Environ Microbiol 24:2078–2088. doi:10.1111/1462-2920.15960. 35254711 PMC9313550

[19] Giglio R, Fani R, Isticato R, De Felice M, Ricca E, Baccigalupi L. 2011. Organization and evolution of the *cotG* and *cotH* genes of *Bacillus subtilis*. J Bacteriol 193:6664–6673. doi:10.1128/JB.06121-11. 21984783 PMC3232876

[20] Naclerio G, Baccigalupi L, Zilhao R, De Felice M, Ricca E. 1996. *Bacillus subtilis* spore coat assembly requires cotH gene expression. J Bacteriol 178:4375–4380. doi:10.1128/jb.178.15.4375-4380.1996. 8755863 PMC178202

[21] Zilhão R, Serrano M, Isticato R, Ricca E, Moran CP, Henriques AO. 2004. Interactions among CotB, CotG, and CotH during assembly of the *Bacillus subtilis* spore coat. J Bacteriol 186:1110–1119. doi:10.1128/JB.186.4.1110-1119.2004. 14762006 PMC344205

[22] Isticato R, Esposito G, Zilhão R, Nolasco S, Cangiano G, De Felice M, Henriques AO, Ricca E. 2004. Assembly of multiple CotC forms into the *Bacillus subtilis* spore coat. J Bacteriol 186:1129–1135. doi:10.1128/JB.186.4.1129-1135.2004. 14762008 PMC344207

[23] Isticato R, Pelosi A, Zilhão R, Baccigalupi L, Henriques AO, De Felice M, Ricca E. 2008. CotC-CotU heterodimerization during assembly of the *Bacillus subtilis* spore coat. J Bacteriol 190:1267–1275. doi:10.1128/JB.01425-07. 18065538 PMC2238189

[24] Black EP, Koziol-Dube K, Guan D, Wei J, Setlow B, Cortezzo DE, Hoover DG, Setlow P. 2005. Factors influencing germination of *Bacillus subtilis* spores via activation of nutrient receptors by high pressure. Appl Environ Microbiol 71:5879–5887. doi:10.1128/AEM.71.10.5879-5887.2005. 16204500 PMC1265928

[25] Cangiano G, Sirec T, Panarella C, Isticato R, Baccigalupi L, De Felice M, Ricca E. 2014. The *sps* gene products affect the germination, hydrophobicity, and protein adsorption *of Bacillus subtilis* spores. Appl Environ Microbiol 80:7293–7302. doi:10.1128/AEM.02893-14. 25239894 PMC4249184

[26] Sirec T, Strazzulli A, Isticato R, De Felice M, Moracci M, Ricca E. 2012. Adsorption of β-galactosidase of *Alicyclobacillus acidocaldarius* on wild type and mutants spores of *Bacillus subtilis*. Microb Cell Fact 11:100. doi:10.1186/1475-2859-11-100. 22863452 PMC3465195

[27] Takamatsu H, Chikahiro Y, Kodama T, Koide H, Kozuka S, Tochikubo K, Watabe K. 1998. A spore coat protein, CotS, of *Bacillus subtilis* is synthesized under the regulation of s^K^ and GerE during development and is located in the inner coat layer of spores. J Bacteriol 180:2968–2974. doi:10.1128/JB.180.11.2968-2974.1998. 9603889 PMC107266

[28] Sambrook J, Fritsch EF, Maniatis T. 1989. Molecular cloning: a laboratory manual, 2nd ed. Cold Spring Harbor Laboratory Press, Cold Spring Harbor, NY.

[29] Cutting S, Vander Horn PB. 1990. Molecular biological methods for *Bacillus*. Wiley, Chichester, United Kingdom.

[30] Steinmetz M, Richter R. 1994. Plasmids designed to alter the antibiotic resistance expressed by insertion mutations in *Bacillus subtilis*, through in vivo recombination. Gene 142:79–83. doi:10.1016/0378-1119(94)90358-1. 8181761

[31] Horton RM, Hunt HD, Ho SN, Pullen JK, Pease LR. 1989. Engineering hybrid genes without the use of restriction enzymes: gene splicing by overlap extension. Gene 77:61–68. doi:10.1016/0378-1119(89)90359-4. 2744488

[32] Maia AR, Reyes-Ramírez R, Pizarro-Guajardo M, Saggese A, Ricca E, Baccigalupi L, Paredes-Sabja D. 2020. Nasal immunization with the C-terminal domain of Bcla_3_ induced specific IgG production and attenuated disease symptoms in mice infected with *Clostridioides difficile* spores. Int J Mol Sci 21:6696. doi:10.3390/ijms21186696. 32933117 PMC7555657

