# Supplementary material for: CotG Mediates Spore Surface Permeability in Bacillus subtilis
Source: mBio. 2022 Nov 10;13(6):e02760-22. doi: 10.1128/mbio.02760-22 (PMC9765600; doi:10.1128/mbio.02760-22)
Supplement: FIG S1 [file mbio.02760-22-s0001.pdf]

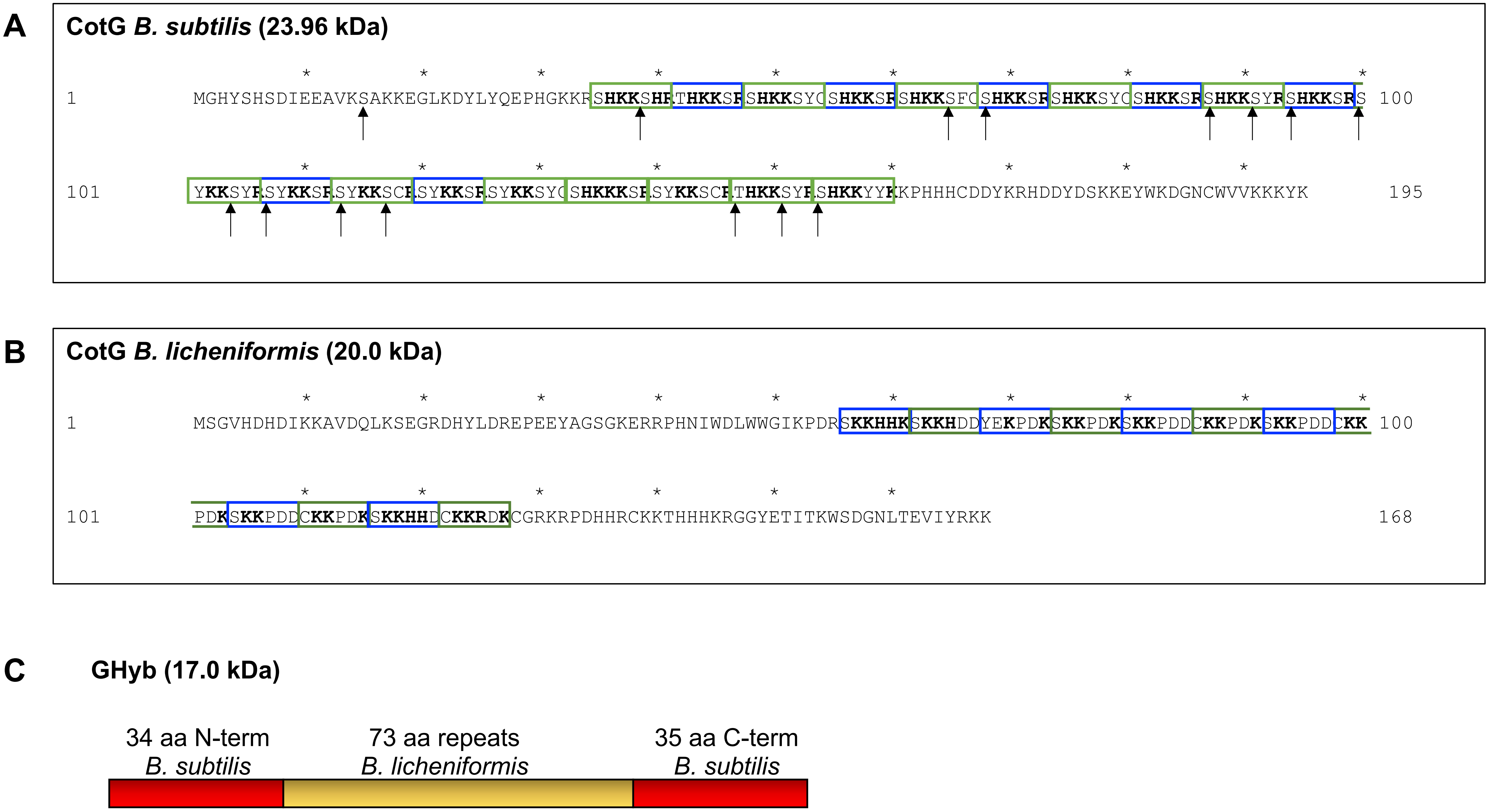

Fig. S1. Primary sequence of CotG in (A) *B. subtilis* PY79 and *B. licheniformis* ATCC14580 (B). Black arrows indicate the sites of phosphorylation in *B. subtilis* CotG. Blue and green rectangles indicate the tandem repeats modules present in CotG. (C) Schematical representation of the fusion protein GHyb.

Fig. S1
