# Supplementary material for: CotG Mediates Spore Surface Permeability in Bacillus subtilis
Source: mBio. 2022 Nov 10;13(6):e02760-22. doi: 10.1128/mbio.02760-22 (PMC9765600; doi:10.1128/mbio.02760-22)
Supplement: FIG S2 [file mbio.02760-22-s0002.pdf]

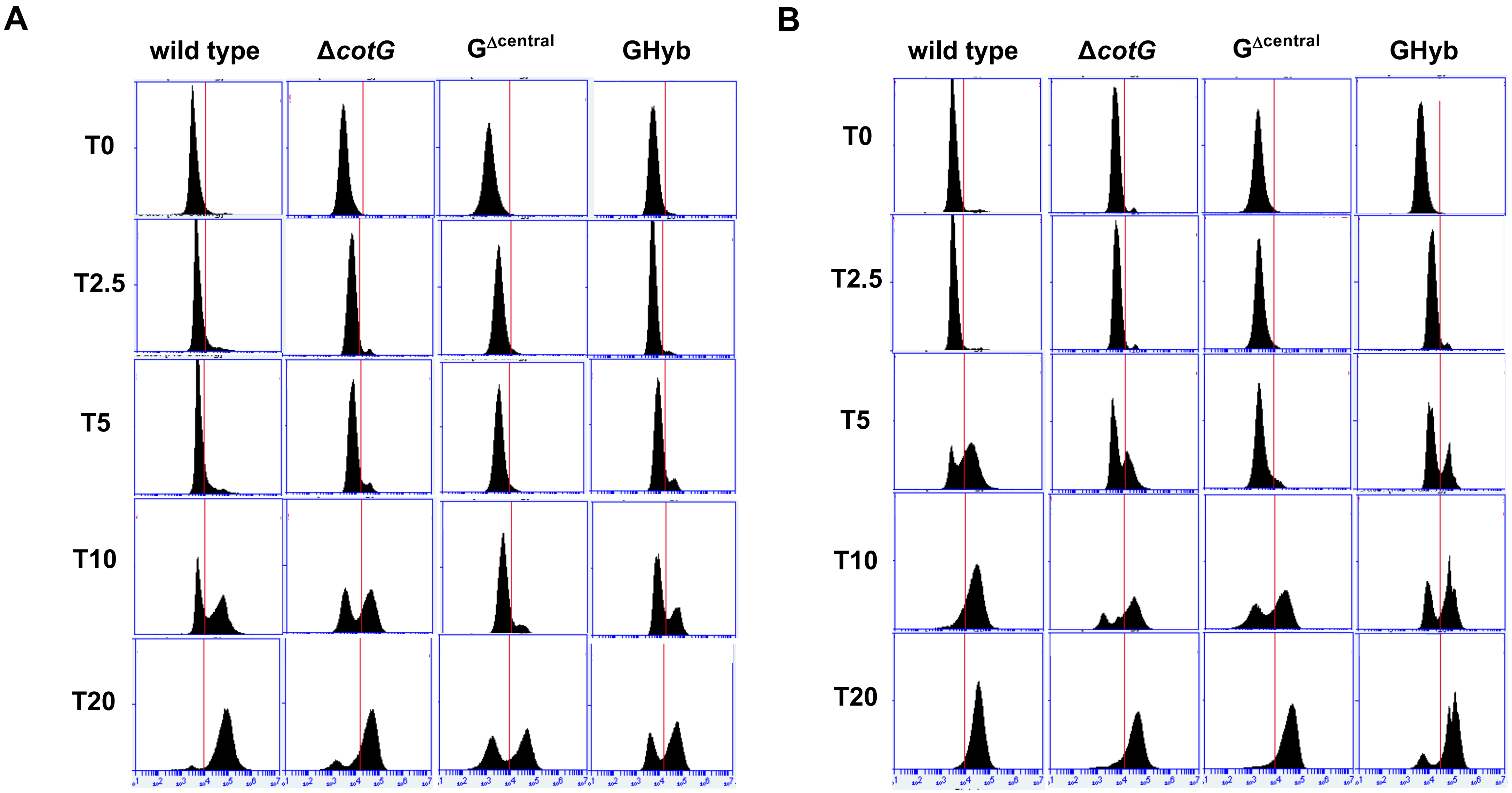

Fig. S2. Germination efficiency determined by flow cytometry using either Asn (A) or Ala (B) as germinant. A similar number (ranging between 35,000 and 38,000) of dormant spores were considered for each strain and fluorescence measured before (T0) and at time intervals after the induction of germination. For each time point of all strains the number of germination-specific fluorescent cells (on the right side of the dotted lines) was counted, and the percentage of germination calculated using the initial number of spores as 100.
