# Supplementary material for: CotG Mediates Spore Surface Permeability in Bacillus subtilis
Source: mBio. 2022 Nov 10;13(6):e02760-22. doi: 10.1128/mbio.02760-22 (PMC9765600; doi:10.1128/mbio.02760-22)
Supplement: TABLE S1 [file mbio.02760-22-s0003.docx]

**Table S1. Presence of *cotC*-*cotU* genes in CotG-like containing *Bacillus* species*.***

| **Species (strain)** | ***cotC* (% identity)** | ***cotU* (% identity)** |
| --- | --- | --- |
| *B. anthracis* (Sterne) | NO | NO |
| *B. cereus* (ATCC 10987) | NO | NO |
| *B. cytotoxicus* (NVH39198) | NO | NO |
| *B. licheniformis* (ATCC 14580) | NO | NO |
| *B. megaterium* (QM B1551) | NO | NO |
| *B. pumilus* (SAFR032) | NO | NO |
| *B. thuringiensis* (ND) | NO | NO |
| *B. weihenstephanensis* (ND) | NO | NO |
| *B. mycoides* (ND) | NO | NO |
| *B toyonensis* (ND) | NO | NO |
| *Bacillus sp.* (1NLA3E) | NO | NO |
| *B. smithii (*ND) | NO | NO |
| *B. bombysepticus* (ND) | NO | NO |
| *Bacillus sp.* (JS) * | YES (78.46) | YES (70.0) |
| *Bacillus sp*. (YP1) * | YES (95.02) | YES (98.85) |

* Strain name is not available (1)
