# Supplementary material for: CotG Mediates Spore Surface Permeability in Bacillus subtilis
Source: mBio. 2022 Nov 10;13(6):e02760-22. doi: 10.1128/mbio.02760-22 (PMC9765600; doi:10.1128/mbio.02760-22)
Supplement: TABLE S2 [file mbio.02760-22-s0004.docx]

**Table S2. *Bacillus* strains used in this study**

| **Species** | **Strains** | **Relevant genotype** | **Reference** |
| --- | --- | --- | --- |
| *Bacillus subtilis* | PY79 | Wild type | (1) |
|  | AZ603 | *ΔcotG ΔcotH::neo* | (2) |
|  | AZ604 | *ΔcotG ΔcotH::neo amyE::cotG_stop_cotH* | (2) |
|  | AZ607 | *ΔcotG ΔcotH::neo amyE::cotG* | (2) |
|  | AZ612 | *ΔcotG ΔcotH::neo thrC::cotGΔ* | (3) |
|  | AZ613 | *ΔcotG ΔcotH::neo* *amyE::cotG_stop_-cotH thrC::cotGΔ* | (3) |
|  | RH238 | *cotC::gfp* (Cm) | (4) |
|  | AZ640 | *cotC::gfp* (Tet L) | This work |
|  | AZ644 | *cotS::gfp* (Cm) | (3) |
|  | AZ583 | *cotS::gfp* (Tet L) | This work |
|  | AZ573 | *cotZ::gfp* (Cm) | (5) |
|  | AZ628 | *cotZ::gfp* (Tet L) | This work |
|  | AZ715 | *thrC::cotGHyb* | This work |
|  | AZ716 | *ΔcotG ΔcotH::neo thrC::GHyb* | This work |
|  | AZ717 | *ΔcotG ΔcotH::neo amyE::cotG_stop_cotH thrC::GHyb* | This work |
|  | AZ742 | *ΔcotG ΔcotH::neo amyE::cotG_stop_cotH thrC::GHyb cotC::gfp* | This work |
|  | AZ751 | *ΔcotG ΔcotH::neo amyE::cotG_stop_cotH thrC::GHyb cotZ::gfp* | This work |
|  | AZ752 | *ΔcotG ΔcotH::neo amyE::cotG_stop_cotH thrC::GHyb cotS::gfp* | This work |
|  | AZ681 | *ΔcotG ΔcotH::neo amyE::cotG_stop_cotH cotS::gfp* | This work |
|  | AZ665 | *ΔcotG ΔcotH::neo amyE::cotG_stop_cotH cotC::gfp* | This work |
|  | AZ682 | *ΔcotG ΔcotH::neo amyE::cotG_stop_cotH cotZ::gfp* | This work |
|  | AZ694 | *ΔcotG ΔcotH::neo amyE::cotG_stop_cotH* thrC::cotGΔ *cotS::gfp::* | This work |
|  | AZ695 | *ΔcotG ΔcotH::neo amyE::cotG_stop_cotH* thrC::cotGΔ *cotZ::gfp* | This work |
|  | AZ696 | *ΔcotG ΔcotH::neo amyE::cotG_stop_cotH* thrC::cotGΔ *cotS::gfp* | This work |
|  | RH201 | *cotB::spc* | (6) |
|  | ER220 | *ΔcotH::spc* | (7) |
|  | AZ664 | *ΔcotH::neo* | This work |
|  | AZ757 | *ΔcotH::neo thrC::cotGHyb,* | This work |
|  | AZ758 | *ΔcotG ΔcotH::neo* thrC::GHyb, *cotC::gfp* | This work |
|  | AZ759 | *ΔcotH::neo thrC::cotGHyb, cotC::gfp* | This work |
|  | AZ760 | *thrC::cotGHyb, cotC::gfp* | This work |
|  | AZ636 | *ΔcotG ΔcotH::neo cotC::gfp* | (8) |
|  | AZ623 | *ΔcotG ΔcotH::neo amyE::cotG* | This work |
| *Bacillus licheniformis* | ATCC14500 | *Wild type* | (9) |

**References:**

1. Youngman P, Perkins JB, Losick R. 1984. A novel method for the rapid cloning in *Escherichia coli* of *Bacillus subtilis* chromosomal DNA adjacent to Tn917 insertions. *Mol Gen Genet* 195:424–433.
2. Saggese A, Scamardella V, Sirec T, Cangiano G, Isticato R, Pane F, Amoresano A, Ricca E, Baccigalupi L. 2014. Antagonistic role of CotG and CotH on spore germination and coat formation in *Bacillus subtilis*. *PLoS One* 9:e104900.
3. Saggese A, Scamardella V, Sirec T, Cangiano G, Isticato R, Pane F, Amoresano A, Ricca E, Baccigalupi L. 2014. Antagonistic role of CotG and CotH on spore germination and coat formation in *Bacillus subtilis*. *PLoS One* 9:e104900.
4. Petrillo C, Castaldi S, Lanzilli M, Saggese A, Donadio G, Baccigalupi L, Ricca E, Isticato R. 2020. The temperature of growth and sporulation modulates the efficiency of spore-display in *Bacillus subtilis*. *Microb Cell Fact* 19:185.
5. Isticato R, Sirec T, Giglio R, Baccigalupi L, Rusciano G, Pesce G, Zito G, Sasso A, De Felice M, Ricca E. 2013. Flexibility of the programme of spore coat formation in *Bacillus subtilis*: bypass of CotE requirement by over-production of CotH. *PLoS One* 8:e74949.
6. Isticato R, Ricca E, Baccigalupi L. 2019. Spore Adsorption as a Nonrecombinant Display System for Enzymes and Antigens. *J Vis Exp* https://doi.org/10.3791/59102.
7. Naclerio G, Baccigalupi L, Zilhao R, De Felice M, Ricca E. 1996. *Bacillus subtilis* spore coat assembly requires cotH gene expression*. J Bacteriol* 178:4375–4380.
8. Donadio G, Lanzilli M, Sirec T, Ricca E, Isticato R. 2016. Localization of a red fluorescence protein adsorbed on wild type and mutant spores of *Bacillus subtilis*. *Microb Cell Fact* 15:153.
9. Saggese A, Isticato R, Cangiano G, Ricca E, Baccigalupi L. 2016. CotG-Like Modular Proteins Are Common among Spore-Forming Bacilli. *J Bacteriol* 198:1513–1520.
