# Supplementary material for: CotG Mediates Spore Surface Permeability in Bacillus subtilis
Source: mBio. 2022 Nov 10;13(6):e02760-22. doi: 10.1128/mbio.02760-22 (PMC9765600; doi:10.1128/mbio.02760-22)
Supplement: TABLE S3 [file mbio.02760-22-s0005.docx]

**Table S3. Oligonucleotides used in this study**

| **Primer** | | **Sequence 5' - 3'** | **Restriction site** | **Position of annealing^(c)^** | **Position of annealing ^(d)^** |
| --- | --- | --- | --- | --- | --- |
| X2 | ggatccGCCTTTATCGTTAGGAT^(a)^ | BamHI | -891/-874(*cotH*) | - |  |
| H19 | aagcttCCATAATCCTCCTTACAAATT^(a)^ | HindIII | -142/-121 (*cotG*) | - |  |
| Glich1 | gcttttatgatgttttttgCTGCGTTTTTTTCCATGAGGC^(b)^ |  | +84/+104 (*cotG*) | +167/+186 |  |
| Glich2 | GCCTCATGGAAAAAAACGCAGcaaaaaacatcataaaagc^(b)^ |  | +84/+104 | +167/+186 |  |
| Glich3 | GCAGTGGTGGTGCGGTTTtttgtcgcgttttttgcag^(b)^ |  | +477/+497 | +362/+381 |  |
| Glich4 | ctgcaaaaaacgcgacaaaAAACCGCACCACCACTG^(b)^ |  | +477/+497 | +362/+381 |  |

(a)Capital and lowercase letters indicate bases of DNA and of an unpaired tail carrying a restriction site, respectively. (b) Lowercase letters in Glich_1_, Glich_2_, Glich_3_ and Glich_4_ indicate sequence derived from *cotG* of *B. licheniformis* ATCC14580. (c) Considering as +1 the first base of the first codon in *cotH* or *cotG* of *B. subtilis* PY79 as indicated in brackets. (d) Considering as +1 the first base of the first codon in *cotG* of *B. licheniformis* ATCC14580 as indicated in brackets. The symbol “- “indicates the absence of the position of annealing.
